# Incidence and determinants of new AIDS-defining illnesses after HAART initiation in a Senegalese cohort

**DOI:** 10.1186/1471-2334-10-179

**Published:** 2010-06-19

**Authors:** Pierre De Beaudrap, Jean-François Etard, Assane Diouf, Ibrahima Ndiaye, Guèye Fatou N Ndèye, Papa S Sow, Kane Coumba T Ndèye, René Ecochard, Eric Delaporte

**Affiliations:** 1 Institut de Recherche pour le Développement (IRD); Université Montpellier1; UMR 145, Montpellier, F-34000, France; 2 Hospices Civils de Lyon, Service de Biostatistique, Lyon, F-69003, France; Université de Lyon, Lyon, F-69000, France; Université Lyon I, Villeurbanne, F-69100, France; CNRS, UMR5558, Laboratoire de Biométrie et Biologie Evolutive, Equipe Biotatistique Santé, Villeurbanne, F-69100, France; 3 Fann University Teaching Hospital, Regional Research and Training Centre for HIV/AIDS, Dakar, Senegal; 4 Fann University Teaching Hospital, Ambulatory Care Unit, Dakar, Senegal; 5 Fann University Teaching Hospital, Department of Infectious Diseases, Dakar, Senegal; 6 Le Dantec Teaching Hospital, Laboratory of Bacteriology and Virology, Dakar, Senegal

## Abstract

**Background:**

Although a dramatic decrease in AIDS progression has been observed after Highly Active Anti Retroviral Therapy (HAART) in both low- and high-resource settings, few data support that fact in low-resource settings.

This study describes the incidence of AIDS-defining illnesses (ADI) after HAART initiation and analyzes their risk factors in a low-resource setting. A focus was put on CD4 cell counts and viral load measurements.

**Methods:**

404 HIV-1-infected Senegalese adult patients were enrolled in a prospective observational cohort and data censored as of April 2008. A Poisson regression was used to model the incidence of ADIs over two periods and to assess its association with baseline variables, current CD4, current viral load, CD4 response, and virological response.

**Results:**

ADI incidence declined from 20.5 ADIs per 100 person-years, 95% CI = [16.3;25.8] during the first year to 4.3, 95% CI = [2.3;8.1] during the fourth year but increased afterwards. Before 42 months, the decrease was greater in patients with clinical stage CDC-C at baseline and with a viral load remaining below 1000 cp/mL but was uniform across CD4 strata (p = 0.1). After 42 months, 293 patients were still at risk. The current CD4 and viral load were associated with ADI incidence (decrease of 21% per 50 CD4/mm^3 ^and of 61% for patients with a viral load < 1000 cp/mL).

**Conclusions:**

During the first four years, a uniform decline of ADI incidence was observed even in patients with low CD4-cell counts at HAART initiation as long as the viral load remained undetectable. An increase was noted later in patients with immunologic and virological failures but also in patients with only virological failure.

## Background

Since the advent of Highly Active Anti Retroviral Therapy (HAART), a dramatic decrease in AIDS progression has been observed in both developed [1-5] and developing countries [6,7]. However, some differences still exist. For instance, the nature of the most frequent AIDS-defining illnesses (ADI) differs between low-resource and industrialized countries [8]; tuberculosis and recurrent bacterial infections are most often observed in the former setting than in the latter [6,7,9,10]. Therefore, results from high-income countries cannot be unreservedly used in low-income ones.

Observational studies have identified the CD4 cell count and the history of AIDS as the strongest predictors of disease progression [5,7,11-18]. The viral load and the virological response (change in the viral load) were also found associated with disease progression [10,19], but this association was less often studied and sometimes found to be weak [20]. Whereas the CD4 cell count is undoubtedly a key marker in monitoring the response to treatment in low-resource settings [21], the place of viral load testing is still under debate [22-24] especially because it is expensive and its feasibility and benefits in such settings are not yet demonstrated. Therefore, the evaluation of the relationships between these longitudinal markers and the occurrence of ADI is important to determine the markers' practical utility.

To date, there are few studies about disease progression in low-resource settings and most have short follow-up durations. Nevertheless, as the access to antiretroviral therapy in such settings is scaled up, there is a need for further knowledge on long-term outcomes in patients put on HAART and for evaluation of clinical or biological markers for patient monitoring. The present study describes the incidence and nature of the most common ADI in a low resource setting and examines the relationships between clinical and biological markers and the occurrence of ADI.

## Methods

### Study design

From August 1998 to April 2002, 404 HIV-1 infected patients aged 15 or more and participating in the "Initiative Sénégalaise d'Accès aux médicaments Antirétroviraux" (ISAARV) were enrolled in an observational cohort after giving written informed consent. The data were censored either at the last visit before April 2008 or at the date of death.

The initial antiretroviral therapy regimen was a triple drug combination (two nucleoside reverse transcriptase inhibitors (NRTI) + either one non-nucleoside reverse transcriptase inhibitor (NNRTI) or one protease inhibitor (PI)), except for 18 patients who received only two NRTI until May 2000. Antiretroviral drugs were provided for free starting from December 2003.

After comprehensive clinical and biological assessments at inclusion, patients were examined at least every 2 months and had a biological evaluation at least every 6 months. A patient record and a Case Report Form that includes a comprehensive list of various ADIs were made available to the investigator at each patient visit.

Patients' monitoring details, characteristics at baseline, antiretroviral treatment efficacy, adherence to treatment, and mortality pattern (early or late) have been previously published [25-28]. The study was approved by the Senegalese National Committee for Health Research (CNRS).

### Outcomes

The primary outcome for the present analysis was the occurrence of a new CDC stage C event (ADI) as defined by the CDC revised classification of 1993 [8]. Only ADIs occurring after HAART initiation were considered. Because it is sometimes difficult to tell whether an event is a relapse or a first episode, only the first event of each ADI was considered in the analysis.

### Laboratory tests

CD4 cell counts were obtained using the FACSCount System (Becton Dickinson, San Jose, CA, USA). Plasma viral loads were obtained using either Amplicor HIV-1 1.5 or 2.0 assay (Roche Molecular Systems, Belleville, NJ, USA) or Bayer bDNA HIV-1 Quantiplex assay 2.0 or 3.0 (Bayer Diagnostics, Tarrytown, NY, USA). All the tests were carried out at Dakar, Senegal.

### Statistical analysis

The ADI incidence rate was estimated for each of seven successive periods (year 1 to 6 and rest of follow-up). That rate was defined as the number of new ADIs divided by the number of person-years (PYs) at risk; i.e., the time accrued from the beginning of the period at risk till the end of that period or till censoring. When an ADI occurred, the patient was considered still at risk for occurrence of other ADIs of other types; ADIs of the same type were excluded.

Incidence rates were also computed for three CD4 cell count categories (less than 200 cells/mm^3^, 200 to 350 cells/mm^3^, and more than 350 cells/mm^3^). Then the number of PYs was counted starting from the date of a CD4 cell count till the first event of the following: next CD4 cell count, new ADI, death, or end of follow-up.

Owing to the seeming change in the incidence rate of ADI, the follow-up was split into two periods: the first period encompassed the first 42 months after HAART initiation and the second period the remaining follow-up.

A Poisson regression was used in each period to model the incidence of ADIs over time and to assess the association between the available variables and the occurrence of ADIs. Confidence intervals were computed using robust variances (sandwich estimators) that take into account the correlation between multiple events within each individual [29]. All models were checked using global goodness of fit statistics as well as various diagnostic plots.

The analyses were carried out as follows:

In a first step, only demographic variables (age, sex) and variables measured at baseline (CD4 cell count, BMI, history of AIDS, haemoglobin, and cotrimoxazole prophylaxis) were included in the analysis. In a second step, the association between the incidence rate of ADI and the current values of the biological markers (CD4 cell count and viral load) was assessed. Viral load was considered as a binary covariate using the threshold 1,000 copies/mL. Lastly, covariates that summarize various aspects of the CD4 and virological response over each period were also included in the analysis. More specifically, the achievement of a viral load below 1,000 cp/mL before 12 months for the first period, the achievement of a viral load below 1,000 cp/mL at 42 months for the second period, the duration of follow-up with a viral load below 1,000 cp/mL and the duration with a viral load above 1,000 cp/mL were considered as summary variables for the virological response. The two latter variables were time-dependent. The changes in CD4 cell count over time were modelled independently of the model for ADIs using random-effects regression of CD4 with time [30]. In such models, the intercept and slope are made up of a fixed effect that represents the average change in CD4 cell count within the cohort, and a random effect that represents the individual deviation from that population average. The random slope was then used as a summary covariate of the CD4 response.

Our analysis focused on the occurrence of ADIs during HAART; thus, the patients were right censored at death. However, death could not be regarded as independent of the occurrence of ADIs because this could lead to biased results [31]. This dependent right-censoring was taken into account by weighting the models with the inverse probability of surviving [32,33]. Irregularly missing variables were imputed under the assumption of missingness at random [34,35]. At baseline, less than 3% of the data were missing but for the viral load (18%) and no systematic pattern was found. A detailed analysis of the missingness in the immuno-virological markers is given elsewhere [36].

The statistical analyses were done with the open access software R [37].

## Results

### Patients' characteristics

At HAART initiation, more than half of the patients (55%) had already experienced an ADI. Almost all patients (95%) were antiretroviral-naive, 78% were receiving cotrimoxazole chemoprophylaxis and 169 (44%) started with a PI-containing regimen. The other characteristics of the patients are summarized in Table 1. During the study period, 106 deaths occurred of whom 15 were due to tuberculosis and 37 (35%) occurred in patients who experienced at least one ADI after HAART initiation.

**Table 1:** Characteristics of the HIV-1 infected participants at HAART initiation (Senegalese cohort, 1998-2008, 404 patients).

Characteristic	n	Value at baseline
Age, median [IQR], (years)	404	37 [31-43]
Gender (Female)	404	
Male		183 (45.3%)
Female		221 (54.7%)
Clinical stage (CDC) C	404	224 (55.5%)
Body mass index, Median [IQR] (kg/m^2^)	391	19.8 [17.9-22.4]
ART naive	383	362 (94.5%)
Haemoglobin level, Median [IQR], (g/dL)	399	10.7 [9.5-12.0]
CD4 cell count, Median [IQR], (cells/μL)	395	128 [54-217]
HIV-1 viral load, Median [IQR], (log cp/mL)	330	5.2 [4.7-5.6]

IQR: interquartile range

The median CD4 cell count increased from 128 cells/mm^3 ^(Interquartile range, IQR: [54;217]) at baseline to 286 cells/mm^3 ^(IQR: [202;413]) at 12 months and to 462 cells/mm^3 ^(IQR: [325;615]) at 72 months.

### AIDS-defining illnesses

At the end of April 2008, 157 first ADIs were reported. Because of concomitant events, the final number of ADIs considered in the analyses was 155 (Table 2). The total follow-up time accrued to 1,575 PYs with a median individual follow-up of 57 months [range: 1-107]. During the study period, 121 patients (30%) experienced at least one CDC-C classifying event. The number of ADIs per individual ranged from 0 to 3.

**Table 2:** AIDS-defining events occurring after HAART initiation, adult Senegalese cohort, 1998-2008 (n = 404).

CDC-C events	N	%
Recurrent bacterial pneumonia	47	30.3
*Mycobacterium tuberculosis *infection (pulmonary or extra-pulmonary)	33	21.3
Chronic herpes simplex	27	17.4
Oesophageal or pulmonary candidiasis	17	11
Kaposi sarcoma	8	5.2
Wasting syndrome	7	4.5
Chronic isosporiasis	4	2.6
Chronic cryptosporidiosis	3	1.9
HIV encephalopathy	2	1.3
Extra-pulmonary cryptococcosis	2	1.3
*Pneumocystis jirovecii *pneumonia	2	1.3
Cerebral toxoplasmosis	1	0.7
Cytomegalovirus retinitis	1	0.7
Other CDC-C disease	1	0.7
Total	155	100

The main ADIs were, in decreasing order, recurrent pneumonia (47 cases), tuberculosis (33 cases), recurrent herpetic ulcerations (27 cases) and visceral candidiasis (19 cases) (Table 2).

### Incidence rates

**Figure 1:**
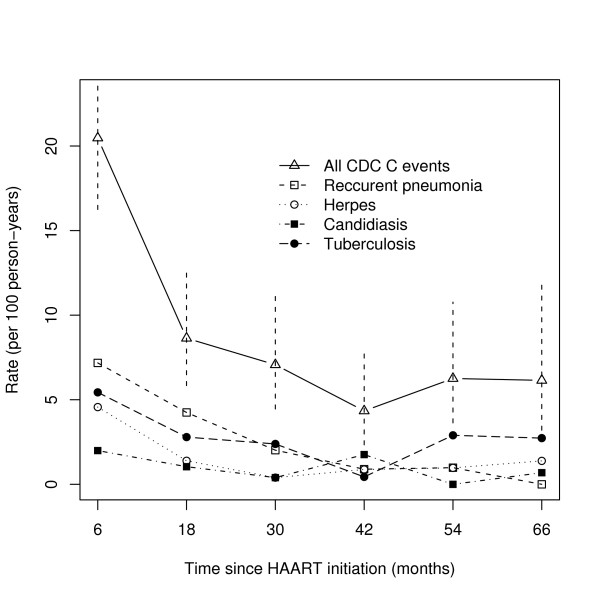
**Incidence rate of AIDS-defining illnesses after HAART plotted against time for: pooled CDC stage C events (with the 95% confidence intervals), recurrent pneumonia, herpes, candidiasis, and tuberculosis (Adult Senegalese cohort, 1998-2008, 404 patients)**.

The incidence rate of ADIs decreased from 20.5 ADIs per 100 PYs (95% CI: [16.3;25.8]) over the first year to 4.3 ADIs per 100 PYs (95% CI: [2.3;8.1]) over the fourth year, which corresponds to an estimated relative decrease in the incidence rate of 5% per month (95% CI: [3;6]) (Figure 1). This significant initial decrease of the incidence rate along time was observed for all ADIs but candidiasis. The monthly relative decrease was 6% (95% CI: [3;9]) for herpes infection, 2% (95% CI: [1;4]) for tuberculosis, and 6% (95% CI: [4;8]) for recurrent pneumonia.

After the fourth year, there was an significant increase of 5% per month in the incidence rate of ADIs (95% CI: [2.1;7.7]).

### Incidence rate by CD4 level

**Figure 2:**
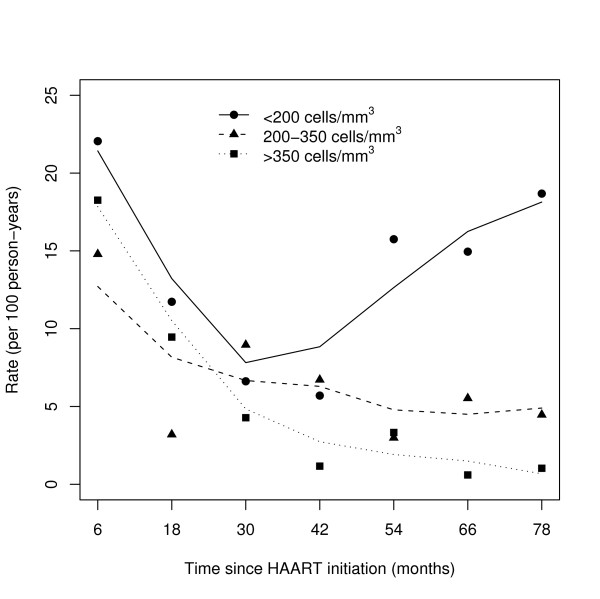
**Incidence rates of AIDS-defining illness after HAART initiation by CD4-cell-count strata: < 200 cells/mm^3^, 200-350 cells/mm^3^, and > 350 cells/mm^3^**. The dark symbols represent the observed values. The lines represent the smoothed curves using the weighted least squares method (Adult Senegalese cohort, 1998-2008, 404 patients).

The incidence rate of ADIs by current-CD4-cell-count stratum is displayed in Figure 2. In patients with less than 200 CD4 cells/mm^3^, the incidence rate of ADIs decreased from 22.0 per 100 PYs (95% CI: [16.5;29.5]) over the first year to 5.7 per 100 PYs (95% CI: [1.4;22.8]) over the fourth year, but increased thereafter to nearly 18 per 100 PYs (95% CI: [7.0;49.8]). In patients with 200 to 350 CD4 cells/mm^3^, the incidence rate of ADIs decreased from 14.8 events per 100 PYs (95% CI: [9.2;28.8]) to 6.7 events per 100 PYs (95% CI: [3.0;15.0]) over the fourth year, then levelled off. Lastly, in patients with more than 350 CD4 cells/mm^3^, a steady decrease in the incidence rate of ADIs from 18.3 events per 100 PYs (95% CI [9.8;34.0]) to 1.3 events per 100 PYs (95% CI [0.5;4.1]) was observed.

### Predictors of ADI during the first period (0-42 months)

During the first period after HAART initiation, the total follow-up accrued to 1,149 PYs and 122 ADIs were observed. The ADI incidence rate was estimated to be as high as 27.4 events per 100 PY at HAART initiation (95% CI [19.5;38.7]) then decreased linearly by 5% per month (95% CI = [2;8]).

In the multivariate analysis, considering only the baseline covariates, the initial incidence rate was twice as high in patients with CDC-C at baseline than in patients with a clinical stage CDC-A or -B (95% CI = [1.40;3.06]). However, the changes in the ADI incidence rate were negatively associated with the baseline clinical stage (estimated decrease of 6% per month, 95% CI = [4;9], for CDC stage C and 1% per month, 95% CI = [0;4], for CDC stage A or B).

When time dependent variables were introduced in the analysis together with the clinical stage at baseline, the change in the ADI incidence was associated with the duration with a viral load below 1,000 cp/mL (5% decrease in the monthly rate of ADI per month with a viral load below 1000 cp/mL, 95% CI = [1;8]). Note here that the rate of decrease in ADI incidence was homogeneous across CD4 strata (p = 0.1).

### Predictors of ADI during the second period (42 months and up)

Only 293 patients were followed-up beyond 42 months and therefore included in this analysis. Their follow-up accrued to 932 PYs and 33 new ADIs occurred during this period.

At 42 months, 165 (56%) patients had a viral load below 1,000 cp/mL and the median time with viral load persistently below 1000 cp/mL was 12 months (IQR = [6;24]).

A linear increase in the CD4 cell count over time was observed (+1.01 CD4/mm^3^/month, 95% CI = [0.41;1.60]) with a large variability in the CD4 slope. Among the 21 patients whose CD4 cell counts decreased below 200 cells/mm^3^, 16 (75%) had a viral load persistently above 1,000 cp/mL.

In the univariate analysis, the incidence rate of new ADI was associated with the achievement of a viral load below 1,000 cp/mL at the start of the period, the current viral load, the duration with a viral load below 1,000 cp/mL, the current CD4 cell count, and the individual variability in the CD4 slope (Table 3). In the multivariate analysis, the incidence rate was negatively associated with the current CD4 cell count (decrease of 21% in the ADI incidence per 50 CD4/mm^3 ^increment, 95% CI = [13;29]) and with a current viral load below 1,000 cp/mL (decrease of 61% in the ADI incidence in patients with a viral load below 1,000 cp/mL compared to those with a viral load above 1,000 cp/mL, 95% CI = [12;83]). No significant interaction between these two variables was observed. Of note, the substitution of variable "current viral load" by variable "duration with a viral load below 1,000 cp/mL" led to an equivalent model.

**Table 3:** Rate ratio relative to the occurrence of AIDS-defining illnesses 42 months after HAART initiation (Adult Senegalese cohort, 1998-2008, 293 patients).

	Univariate analysis		Multivariate analysis	
Factors	Rate ratio	95% CI	Rate ratio	95% CI
Age (year)	1.00	[0.96;1.05]		
Sex (female vs. male)	0.52	[0.25; 1.07]		
Baseline CD4^a^	0.95	[0.82;1.10]		
Baseline BMI^b ^(kg/m^2^)	0.90	[0.80; 1.01]		
CDC stage at baseline (C vs. A or B)	1.77	[0.77;4.09]		
VL^c ^≤ 10^3 ^cp/mL at period start	0.33	[0.15;0.73]		
Current CD4 cell count^a^	0.80	[0.74;0.87]	0.79	[0.71;0.87]
Variability in CD4 increase^d^	0.61	[0.44;0.84]		
Current viral load (≤ 10^3 ^vs. > 10^3 ^cp/mL)	4.33	[1.86;10.08]	2.74	[1.15;6.57]
Duration of VL^c ^≤ 10^3 ^cp/mL	0.88	[0.81; 0.96]		

a: per 50 cells/mm^3^; b: Body Mass Index; c: Viral Load; d: per standard deviation unit.

## Discussion

Accurate information on morbidity after HAART initiation in low-resource settings is still needed to improve treatment and monitoring guidelines. The present study presents the incidence of new ADIs in such a setting and the main predictors of ADI occurrence: CD4 cell count and viral load.

The ADI incidence rates observed in the Senegalese cohort were consistent with those seen in other low-resource settings [10,39] but higher than those seen in industrialized settings [5,20]. The most frequent ADIs that occurred during HAART were similar to those seen in other low-resource settings, tuberculosis being among the leading ones [6,7,10]; its estimated incidence in the Senegalese cohort was comparable to that reported in other similar studies [39,40]. Besides, the rate at which the incidence rate of ADIs decreased was different between ADIs, which is also consistent with previous observations [2,5].

A decrease in the ADI incidence rate over the first years after HAART initiation has been observed in other low-resources settings [1,4-7,10,19,41]. Interestingly, similar rates of decrease in the ADI incidence were found among patients with different CD4 levels while these rates were different among patients with different virological responses. This result underlines the importance of the initial virological response. Also, patients who already experienced an ADI before starting HAART had an increased risk of new ADIs during the first four years of treatment, indicating that antiretroviral therapy should be initiated well before the occurrence of a first ADI.

Despite the decrease in the ADI incidence rate during the first four years on HAART, an overall increase was observed afterwards, which was mainly due to the high incidence rate of ADIs in patients with less than 200 CD4 cells/mm^3 ^and a VL persistently above 1,000 cp/mL. Moreover, after four years on HAART, that decrease persisted only in patients with more than 350 CD4 cells/mm^3^. These results indicate that the association between the CD4 cell count and the occurrence of ADIs changes with time (non-proportional effect) and that the CD4 cell count presents an increased predictive value after 4 years on HAART. It should be noted that, during the second period, the current CD4 value was a stronger predictor of ADI than the individual variation in the CD4 slope.

The effect of the virological response on the occurrence of new ADIs could be separated into a direct effect independent of the CD4 response and an indirect effect mediated by the effect of the virological response on the CD4 response. This may explain the two-fold decrease in the rate ratio associated with the current viral load between the univariate (marginal) and the multivariate (conditional) model.

The role of the viral load in monitoring AIDS patients after HAART initiation in low-resource settings is still not sufficiently clear or recognized [22-24]. One finding of the present study is that, along with the CD4 cell count, the viral load was an important predictor of the occurrence of new ADIs, especially during the first years of ART. We have also found that, after 42 months, ADIs could occur in patients who are immunologically stable but have a detectable viral load. These results suggest that the viral load may complement the CD4 cell count in identifying patients with high risks of disease progression. The treatment strategy in such patients should not be limited to a change in antiretroviral drugs but extended to an intensive and comprehensive clinical care that enhances adherence to the treatment.

The present study has several strengths and limitations. Despite its limited size, the study cohort boasts a long follow-up period and few lost to follow-ups. Regular visits with clinical examination allowed frequent information on new ADI occurrence. However, it must be noted that, in our setting, the diagnostic procedures were more limited than in industrialized settings. It is therefore likely that the incidence of some ADIs, such as pneumocystosis, that require advanced diagnostic procedures, could have been underestimated and some misclassification bias introduced. However, those diagnostic procedures did not change over time, protecting from a differential misclassification with time.

Another limitation could have been the lack of information on cause-specific mortality. However, the outcome of this study was restricted to the occurrence of ADIs. The dependence between mortality and morbidity could have biased the estimations of the rate ratios but this was taken into account by weighting the models with the inverse probability of surviving.

## Conclusions

In this cohort, the incidence rate of ADIs decreased uniformly during the first four years on HAART but increased later, mostly in patients with virological failure and a CD4 cell count of less than 200 cells/mm^3^. During the first four years after HAART initiation, the baseline clinical stage and the current viral load (but not the CD4 cell count) were found to be strong predictors of ADI occurrence. Therefore, as time on HAART extends, regular CD4 cell counts and viral load measurements should find an increasing importance in the monitoring of patients living with AIDS.

## Abbreviations

ADIs: AIDS-defining illnesses; BMI: Body Mass Index; HAART: Highly Active Anti Retroviral Therapy; IQR: Interquartile Range; ISAARV: Initiative Sénégalaise d'Accès aux médicaments Antirétroviraux; NRTI: nucleoside reverse transcriptase inhibitor; NNRTI: non-nucleoside reverse transcriptase inhibitor; PI: protease inhibitor.

## Competing interests

The authors declare that they have no competing interests.

## Authors' contributions

PDB participated to the statistical analysis, interpretation of the results and drafted the manuscript. JFE participated to the data analysis, interpretation of the results and revised critically the article. AD, IN, NG and NK participated to the acquisition of data and revised of the article. PS participated to the conception and design of the study and revised the article. RE participated to the data analysis and revised the article. ED participated to the conception and design of the study and revised critically the article. All the authors have read and approved the version of the article to be published.

## Pre-publication history

The pre-publication history for this paper can be accessed here:

http://www.biomedcentral.com/1471-2334/10/179/prepub
